# Variants in genes encoding the SUR1-TRPM4 non-selective cation channel and sudden infant death syndrome (SIDS): potentially increased risk for cerebral edema

**DOI:** 10.1007/s00414-022-02819-9

**Published:** 2022-04-26

**Authors:** Dong Qu, Peter Schürmann, Thomas Rothämel, Thilo Dörk, Michael Klintschar

**Affiliations:** 1 Institute of Legal Medicine, Hannover Medical School, Carl-Neuberg-Str. 1, 30625 Hannover, Germany; 2 Gynaecology Research Unit, Hannover Medical School, Carl-Neuberg-Str. 1, 30625 Hannover, Germany

**Keywords:** SIDS, SUR1/*ABCC8*, *TRPM4*, Single nucleotide polymorphism (SNP), Cerebral edema

## Abstract

Increasing evidence suggests that brain edema might play an important role in the pathogenesis of sudden infant death syndrome (SIDS) and that variants of genes for cerebral water channels might be associated with SIDS. The role of the sulfonylurea receptor 1 (SUR1)–transient receptor potential melastatin 4 (TRPM4) non-selective cation channel in cerebral edema was demonstrated by extensive studies. Therefore, we hypothesized that variants at genes of the SUR1-TRPM4 channel complex might be linked to SIDS. Twenty-four polymorphisms in candidate genes involved in the SUR1-TRPM4 non-selective cation channel were investigated in 185 SIDS cases and 339 controls. One (rs11667393 in *TRPM4*) of these analyzed SNPs reached nominal significance regarding an association with SIDS in the overall analysis (additive model: *p* = 0.015, OR = 1.438, 95% CI = 1.074–1.925; dominant model: *p* = 0.036; OR = 1.468, 95% CI = 1.024–2.106). In the stratified analysis, further 8 variants in *ABCC8* (encoding SUR1) or *TRPM4* showed pronounced associations. However, none of the results remained significant after correction for multiple testing. This preliminary study has provided the first evidence for a genetic role of the SUR1-TRPM4 complex in the etiology of SIDS, and we suggest that our initial results should be evaluated by further studies.

**Supplementary Information:**

The online version contains supplementary material available at 10.1007/s00414-022-02819-9.

## Introduction

Sudden infant death syndrome (SIDS), the leading cause of death in infants aged 1 month to 1 year in developed countries, is the sudden death of an infant under 1 year of age that remains unexplained after a thorough case investigation, including performing a complete autopsy, examining the death scene, and reviewing the clinical history [1, 2]. However, the specific etiologic mechanisms of SIDS are poorly understood. The triple risk model suggests that a combination of a critical developmental stage, a vulnerable infant, and extrinsic factors is associated with SIDS [3].

Previous relevant studies have shown increased brain weight and/or cerebral edema in some cases of SIDS [4–8], although the results of some studies are inconsistent with this [9–11]. For instance, the study conducted by Aranda et al. demonstrated that the cases diagnosed as SIDS had heavier brain weights compared to age-matched controls [4], and the findings were in line with other related studies [6–8]. The elevated brain weight in SIDS is thought to be possibly due to abnormal brain development or cerebral edema [4, 6], whereas the exact pathogenic mechanism is still uncertain. In contrary, Studer et al. reported that they did not find an elevated brain-to-body weight ratio in SIDS cases; however, they also mentioned that cytotoxic edema in the central nervous system (CNS) does not always lead to brain swelling [9]. In several Norwegian studies, genetic variants of aquaporin genes (AQP) like AQP1, 4, and 9, as critical rapid water transport channels on the plasma membrane, were associated with SIDS [12–14]. Especially, aquaporin-4 (AQP4), a main water channel in the central nervous system, is known to have a key role in the formation of cerebral edema [15–17]. For SIDS cases that are carriers of several specific *AQP4* variant genotypes (CT/TT, rs2075575), an increased brain-to-body weight ratio was found, and thus a putative association of *AQP4* variants and cerebral edema in SIDS [12]. In a recent study, the lower expression of AQP4 in the brain tissue was observed in the carriers with the CT/TT genotype of rs2075575 [18].

In addition to constitutively expressed transporters like AQP4, Kir4.1 as an ATP-sensitive K^+^ channel could assemble with AQP4 to form a complex (water-ion transport coupling) that plays an important role in cerebral edema [19]. Opdal et al. demonstrated the potential link between the SNP locus of the gene encoding Kir4.1 and SIDS [14]. Like Kir4.1, the inducible SUR1-TRPM4 non-selective cation channel plays a similar role in the mechanism of brain edema formation when ATP is depleted and Ca^2+^ concentration increases [20–22]. It has been previously observed that AQP4 also could co-associate physically with SUR1-TRPM4 to form a heteromultimeric complex, as ion/water osmotic coupling, which exerts an important role in astrocyte swelling and cerebral edema caused after injury [23]. The SUR1-TRPM4 non-selective cation channel includes two subunits: sulfonylurea receptor 1 (SUR1, encoded by the *ABCC8* gene) is a member of the ATP-binding cassette transporter family, with its activity modulated by the intracellular ATP/ADP ratio [24]; transient receptor potential melastatin 4 (TRPM4) is a Ca^2+^-regulated non-selective ion channel [25]. Preclinical and clinical trials indicate that pharmacological blockade of SUR1-TRPM4 could be used to reduce secondary brain swelling after injury [24, 26]. Additionally, studies have shown an association between intracranial pressure or cerebral edema after brain injury and several genetic SNP loci in these genes (*ABCC8* and *TRPM4*) [27–29].

Therefore, we hypothesized that variants located in the *ABCC8* and *TRPM4* genes may lead to an increased risk to develop cerebral edema and thus infants susceptible to SIDS and investigated 185 SIDS cases and 339 controls for 24 polymorphisms in candidate genes involved in the SUR1-TRPM4 non-selective cation channel relevant to the development of cerebral edema.

## Materials and methods

### Study subjects

The SIDS cohort (*n* = 185) was enrolled from the Institute of Legal Medicine, Hannover Medical School, and its branch (Oldenburg). The enrollment standards based on the San Diego definition of SIDS [1] were briefly as below: (1) unexpected death during the sleep occurring in their first year of life; (2) no specific cause of death diagnosed after thorough postmortem examination, review of the case history, and death scene investigation. In approximately 50% of the cases, toxicological and/or histological examinations were performed, as commissioned by the respective prosecutor’s office. Radiological or specialized neuropathological investigations as well as genetic screening were performed only for very few cases. 62.6% of the SIDS cases were boys. The average age was 130 days. Among the SIDS cases documented with sleeping positions (*n* = 46), 73.9% of cases were prone positions. The control cohort (*n* = 339) comprised 164 males, 146 females, and 29 gender-unrecorded individuals. It comprised 33 children and 306 healthy adults. The 33 included children in controls were in the first year of life but died due to an explicit cause of death but not SIDS (mostly trauma, infections, and congenital heart defects). Owing to anonymization exact age information was not available in controls. The local ethics committee of Hannover Medical School approved this study.

### Selection of SNPs and genotyping assays

The selection of candidate SNPs of *ABCC8* and *TRPM4* was on account of previously published studies in which a potential association with a specific disease or a possible influence on gene expression was reported. In brief, the selection criteria were as follows: (1) the minor allele frequency (MAF) ≥ 0.05 in Caucasians; (2) SNPs were not in high linkage disequilibrium (LD) (*R*^2^ < 0.8); (3) SNPs were reported in published studies or in the GTEx portal (https://www.gtexportal.org/home/) to influence related gene expressions.

A total of 24 candidate SNP loci (14 in the *ABCC8* gene, 10 in the *TRPM4* gene) were included in the analysis. The detailed information of these 24 SNPs is given in Table 1 and Supplementary material 1.

**Table 1:** Detailed information of 24 SNPs included in this study

SNP	Type	Gene	Position (GRch37)	European MAF*	Genotype distribution (XX: XY: YY)	
					SIDS	Control
rs1048099	G > A	*ABCC8*	chr11:17,496,516	A = 0.480	47:94:44	83:177:79
rs10766397	T > C	*ABCC8*	chr11:17,430,648	C = 0.375	Low call rates	
rs11024286	G > A	*ABCC8*	chr11:17,459,107	A = 0.360	78:84:22	153:143:42
rs1799857	G > A	*ABCC8*	chr11:17,452,492	A = 0.460	64:85:36	109:158:72
rs1799859	C > T	*ABCC8*	chr11:17,419,279	T = 0.291	107:63:15	176:142:21
rs2283258	C > T	*ABCC8*	chr11:17,473,437	T = 0.312	115:97:19	161:123:27
rs2283261	A > C	*ABCC8*	chr11:17,460,899	C = 0.403	69:92:24	128:161:50
rs3758953	A > G	*ABCC8*	chr11:17,478,000	G = 0.497	49:95:41	87:165:87
rs3819521	C > T	*ABCC8*	chr11:17,486,737	T = 0.351	84:78:22	156:143:39
rs4148622	G > A	*ABCC8*	chr11:17,449,002	A = 0.260	98:66:20	193:122:23
rs60105962	T > C	*ABCC8*	chr11:17,459,931	C = 0.405	Deviation from HWE	
rs7105832	A > C	*ABCC8*	chr11:17,488,661	C = 0.351	81:84:20	157:143:39
rs7112138	G > A	*ABCC8*	chr11:17,414,013	A = 0.373	72:81:31	140:151:48
rs7950189	C > T	*ABCC8*	chr11:17,468,317	T = 0.454	52:89:44	88:176:75
rs985136	G > C	*ABCC8*	chr11:17,497,794	C = 0.489	43:96:46	89:172:77
rs11083962	T > G	*TRPM4*	chr19:49,663,476	G = 0.486	41:87:55	92:162:85
rs11083963	A > G	*TRPM4*	chr19:49,665,340	G = 0.453	50:96:38	96:165:76
rs11667393	A > G	*TRPM4*	chr19:49,662,027	G = 0.283	80:88:17	179:143:17
rs12980226	A > C	*TRPM4*	chr19:49,665,451	C = 0.321	90:78:17	159:142:38
rs34271662	A > G	*TRPM4*	chr19:49,689,867	G = 0.347	Failed clustering analysis	
rs3760662	A > G	*TRPM4*	chr19:49,660,889	G = 0.443	60:79:49	95:163:75
rs4802581	T > C	*TRPM4*	chr19:49,666,573	C = 0.450	Deviation from HWE	
rs7251160	C > T	*TRPM4*	chr19:49,683,266	T = 0.333	78:85:22	150:144:44
rs8104571	C > T	*TRPM4*	chr19:49,712,908	T = 0.050	Low call rates	

^*^The European minor allele frequency (MAF) was based on the European population data not including the Finnish dataset in the 1000G Project

Alleles X and Y represent major and minor alleles respectively

DNA extraction was performed from blood, saliva, or thymus samples using the QIAamp DNA Mini Kit (Qiagen, Hilden, Germany) following the manufacturer’s instructions. All DNA samples were kept at − 20 °C for long-period storage. As described in our previous studies [30], genotyping was performed using 192.24 dynamic arrays in the Biomark EP1 platform (Fluidigm, South San Francisco, CA, USA), and the original data was analyzed using the Fluidigm SNP Genotyping Analysis Software Version 4. Probes and related primers were listed in Supplementary material 2. Total, three genotyping arrays were run. For quality control, 46 duplicates (8.7% samples from both cases and controls) were included in the genotyping, and two negative controls (no DNA) were assigned in each array.

### Data analyses

Two SNPs (rs10766397 and rs8104571) had low call rates (< 95%) and were excluded for further analyses. Among the remaining 22 SNPs, one SNP (rs34271662) was excluded owing to the failure of clustering. Chi-square (*χ*^2^) tests were applied to check the Hardy–Weinberg equilibrium (HWE) in controls using an online HWE calculator (https://wpcalc.com/en/equilibrium-hardy-weinberg/). Two SNPs (rs4802581 and rs60105962) deviating from HWE were eliminated for the subsequent analysis. For the 19 remaining variants, a 2 × 2 chi-square (*χ*^2^) test or a Fisher exact test was utilized to test the association of SNPs and SIDS using dominant, and recessive models and a linear-by-linear model of the *χ*^2^ test (same to the Cochrane-Armitage trend test) was used under the additive model. Odds ratios (ORs), 95% confidence intervals (CIs), and corresponding *p* values were calculated.

For the stratified analysis, study subjects were categorized into 4 different groups according to the risk factors for SIDS. These were (1) gender (males (*n* = 99), females (*n* = 59)), age group (0–4 months (*n* = 104), 2–4 months (*n* = 75), 4–8 months (*n* = 51), 8–12 months (*n* = 9)), time of death season (spring (*n* = 40), summer (*n* = 30), spring + summer (*n* = 70), autumn (*n* = 35), winter (*n* = 40), autumn + winter (*n* = 75)), and sleeping positions (prone position (*n* = 34), other positions (*n* = 12)) as shown previously [31].

**Fig. 1:**
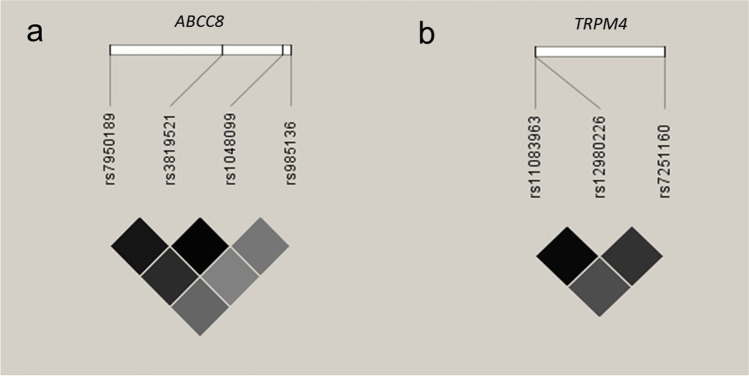
Linkage disequilibrium (LD) estimation among the selected SNPs included in the haplotype blocks of *ABCC8* (**a**) and *TRPM4* (**b**)

SNPs in linkage with each other (*R*^2^ < 0.8 and *D*′ > 0.75) among the European population were included for the haplotype analysis. Haplotype analysis was performed by Haploview 4.2 (Broad Institute, Cambridge, MA, USA) in the study subjects and by LDlink (https://ldlink.nci.nih.gov/) in the European population (Fig. 1). A two-sided *p* value < 0.05 was considered indicating statistical significance in all statistical analyses. Bonferroni correction was used for multiple comparisons, and *p* < 0.0001 was considered statistical significance after multiple testing. All statistical analyses were performed using SPSS 24.0 software (SPSS Inc. Chicago, IL, USA).

## Results

We successfully genotyped 19 variants at *TRPM4* and *ABCC8* in up to 185 SIDS cases and 339 controls. All nominally significant results (*p* < 0.05) of the chi-square analysis are listed in Table 2. In the overall analysis, one SNP (rs11667393 in the *TRPM4* gene) was below the significance threshold of *α* = 0.05 (additive model: *p* = 0.015, OR = 1.438, 95% CI = 1.074–1.925; dominant model: *p* = 0.036; OR = 1.468, 95% CI = 1.024–2.106).

**Table 2:** Selected associations of ABCC8 and TRPM4 polymorphisms with SIDS

Stratum	Gene	SNP	Genotype distribution in SIDS (XX: XY: YY)	*Additive* (Y vs. X alleles)		*Dominant* (YY/XY vs. XX genotypes)		*Recessive* (YY vs. XX/XY genotypes)	
				OR (95% CI)*	*p* value*	OR (95% CI)	*p* value	OR (95% CI)	*p* value
Overall (*n* = 185)	*TRPM4*	rs11667393	80:88:17	1.438 (1.074–1.925)	**0.015 (G)**	1.468 (1.024–2.106)	**0.036 (GG/AG)**	1.916 (0.954–3.846)	0.064
Age 0–4 months (*n* = 103)	*ABCC8*	rs4148622	54:33:16	1.354 (0.976–1.879)	0.069	1.208 (0.776–1.880)	0.403	2.519 (1.420–4.975)	**0.006 (AA)**
Age 2–4 months (*n* = 74)	*ABCC8*	rs4148622	41:22:11	1.255 (0.860–1.830)	0.239	1.071 (0.646–1.778)	0.790	2.392 (1.110–5.155)	**0.022 (AA)**
Age 4–8 months (*n* = 51)	*ABCC8*	rs7105832	15:27:09	1.613 (1.060–2.456)	**0.026 (C)**	2.070 (1.093–3.923)	**0.023 (CC/AC)**	1.647 (0.746–3.650)	0.213
Age 4–8 months (*n* = 51)	*ABCC8*	rs3819521	17:24:10	1.534 (1.011–2.331)	**0.044 (T)**	1.715 (0.922–3.915)	0.086	1.870 (0.868–4.032)	0.105
Age 8–12 months (*n* = 9)	*ABCC8*	rs7950189	5:02:02	0.531 (0.194–1.452)	0.217	0.280 (0.074–1.068)	**0.048 (TT/CT)**	1.006 (0.205–4.950)	0.994
Spring (*n* = 40)	*ABCC8*	rs4148622	14:15:11	2.443 (1.533–3.849)	**0.0004 (A)**	2.472 (1.247–4.910)	**0.008 (AA/AG)**	5.208 (2.304–11.765)	**0.0002 (AA)**
Summer (*n* = 30)	*TRPM4*	rs3760662	14:14:02	0.486 (0.274–0.864)	**0.014 (G)**	0.456 (0.214–0.971)	**0.038 (GG/AG)**	0.246 (0.057–1.055)	**0.042 (GG)**
Summer (*n* = 30)	*TRPM4*	rs11083962	1:16:13	2.387 (1.346–4.234)	**0.003 (G)**	10.802 (1.450–80.432)	**0.004 (GG/GT)**	2.283 (1.066–4.902)	**0.030 (GG)**
Spring + Summer (*n* = 69)	*ABCC8*	rs4148622	32:22:15	1.732 (1.195–2.511)	**0.004 (A)**	1.539 (0.915–2.588)	0.103	3.802 (1.866–7.752)	**0.0002 (AA)**
Spring + Summer (*n* = 70)	*TRPM4*	rs3760662	31:26:13	0.679 (0.470–0.981)	**0.039 (G)**	0.502 (0.296–0.852)	**0.010 (GG/AG)**	0.784 (0.407–1.511)	0.467
Spring + Summer (*n* = 69)	*TRPM4*	rs11083962	11:33:25	1.548(1.071–2.237)	**0.020 (G)**	1.964(0.987–3.906)	0.051	1.698 (0.980–2.941)	0.057
Winter (*n* = 40)	*ABCC8*	rs7112138	10:20:10	1.712 (1.080–2.710)	**0.022 (A)**	2.110 (0.999–4.464)	**0.046 (AA/AG)**	2.021 (0.929–4.405)	0.072
Prone sleep position (*n* = 34)	*ABCC8*	rs3758953	4:16:14	1.810 (1.079–3.038)	**0.025 (G)**	2.589 (0.887–7.559)	0.072	2.028 (0.982–4.184)	0.052
Other sleep positions (*n* = 12)	*ABCC8*	rs7950189	1:05:06	2.721 (1.091–6.783)	**0.032 (T)**	3.857 (0.491–30.303)	0.168	*3.521 (1.104–11.236)*	**0.024 (TT)**

*p* value < 0.05 marked in bold. OR* and *p* values* in the additive model were calculated using the linear-by-linear association of the chi-square test

Alleles X and Y represent major and minor alleles respectively

The stratified analysis was performed to evaluate the effects of the variants located in *ABCC8* and *TRPM4* on the hazards of SIDS by age, gender, time of death season, and sleep positions. In the stratified results, 14 nominally significant results (*p* < 0.05) were obtained. The only one associated locus in the main analysis, rs11667393, showed no specific associations in any one subcategory. However, rs4148622 in *ABCC8* indicated significant differences between cases and controls in four subgroups (“age 0–4 months,” “age 2–4 months,” “Spring,” and “Spring + Summer”), and rs7950189 in *ABCC8* in two subgroups (“age 8–12 months” and “other sleep positions”). Both rs3760662 and rs11083962 in the *TRPM4* gene were associated with SIDS in two subgroups (“Summer” and “Spring + Summer”). Variants rs7105832 and rs3819521 located in *ABCC8* showed associations with SIDS in one subgroup (“age 4–8 months”). In the “Winter” subcategory, rs7112138 from *ABCC8* was indicated to be related to SIDS. SNP rs3758953 at the *ABCC8* gene had a single association in the category “Prone sleep position.” None of the above-mentioned findings in the stratified analysis remained statistically significant after Bonferroni correction at *α* = 0.0001, but these results may still suggest a biological relevance for the etiology of SIDS.

In a haplotype analysis, the extent of LD among the identified SNPs in *ABCC8* and *TRPM4* was estimated using Haploview 4.2 and LDlink. The related haplotype blocks were respectively screened in *ABCC8* and *TRPM4* (Fig. 1). The haplotype block in *ABCC8* was comprised of 4 SNPs (rs7950189, rs3819521, rs1048099, and rs985136). The haplotype-based association analysis provided evidence that the haplotype TCAG in *ABCC8* may be relevant for SIDS (*p* = 0.040) (Table 3). As for *TRPM4*, three SNPs (rs11083963, rs12980226, and rs7251160) were included in the block. *TRPM4* haplotype AAT was significantly associated with SIDS (*p* = 0.031) (Table 4). Nevertheless, both potentially associated risk haplotypes were rare in both SIDS and controls.

**Table 3:** Results of the haplotype analysis of ABCC8 markers rs7950189, rs3819521, rs1048099, and rs985136

Haplotype	Freq	SIDS, control ratio counts	SIDS, control frequencies	*p* value
CCGG	0.391	147.6: 222.4, 262.4: 415.6	0.399, 0.387	0.704
TTAC	0.259	96.7: 273.3, 174.8: 503.2	0.261, 0.258	0.901
TCAC	0.133	48.0: 322.0, 91.4: 586.6	0.130, 0.135	0.816
TTAG	0.059	18.7: 351.3, 43.1: 634.9	0.050, 0.064	0.387
CCGC	0.054	15.8: 354.2, 40.5: 637.5	0.043, 0.060	0.244
TCGC	0.04	14.5: 355.5, 27.7: 650.3	0.039, 0.041	0.898
TCAG	0.023	13.3: 356.7, 10.8: 667.2	0.036, 0.016	**0.040**
CCAC	0.016	4.3: 365.7, 12.7: 665.3	0.012, 0.019	0.385

*p* value < 0.05 marked in bold

**Table 4:** Results of the haplotype analysis of TRPM4 markers rs11083963, rs12980226, and rs7251160

Haplotype	Freq	SIDS, control ratio counts	SIDS, control frequencies	*p* value
GAC	0.482	179.1: 190.9, 326.1: 351.9	0.484, 0.481	0.923
ACT	0.28	102.2: 267.8, 191.5: 486.5	0.276, 0.282	0.830
AAC	0.139	53.2: 316.8, 92.7: 585.3	0.144, 0.137	0.752
GAT	0.044	13.6: 356.4, 32.3: 645.7	0.037, 0.048	0.410
ACC	0.03	6.5: 363.5, 25.0: 653.0	0.018, 0.037	0.081
AAT	0.02	12.1: 357.9, 8.9: 669.1	0.033, 0.013	**0.031**

*p* value < 0.05 marked in bold

To assess a potential functional relevance for the gene expression, cis-eQTL target gene expression was employed using the online data from Genotype-Tissue Expression (GTEx) project. Eight of the nine SNPs with nominally statistical significance (except rs3758953 from *ABCC8*) were reported to influence the gene expression in tissue from the CNS.

## Discussion

It has been postulated that disturbances in the brain water balance may be involved in the pathogenesis of at least some SIDS cases. Previous studies have uncovered that SNP loci for genes involved in ion channels and water channels (like AQP1, 4, 9, and Kir4.1) might be associated with SIDS [12–14], although one study did not confirm this finding [9]. The opening of SUR1-TRPM4 channels is associated with an undifferentiated influx of monovalent cations and cell depolarization, followed by cytotoxic edema, cell death, blood–brain barrier (BBB) breakdown, and formation of vasogenic edema in the CNS, suggesting an important role of the SUR1-TRPM4 non-selective cation channel in brain edema [27–29]. Accordingly, it is reasonable to speculate that this SUR1-TRPM4 complex might play a potential role at least in some subgroups of SIDS, although up to now this has never been studied. In the present study, we thus investigated the genotypes and haplotypes of 24 SNP loci located in the corresponding genes (*ABCC8* and *TRPM4*).

Overall, there was evidence that 9 of the 24 candidate SNP loci could be associated with SIDS, though 8 of them only in subgroups. Three putative risk SNPs were located within *TRPM4* and the other six in *ABCC8*. The only nominally significant SNP in the main analysis was located in *TRPM4*, and the other 8 genetic variants were identified in the stratified analysis (*ABCC8* (6 variants) and *TRPM4* (2 variants)).

The G allele or GG/AG genotypes of polymorphism rs11667393 located in *TRPM4* were associated with SIDS (allele G in SIDS: 32.9%, vs. 26.1% in controls), suggesting that this SNP locus could be an influential predisposing factor. However, in a study on cerebral edema secondary to traumatic brain injury (TBI), no association for this locus was shown [27]. According to the GETx database (although being an intronic variant), carriers of the rs11667393 G allele typically have alerted mRNA levels of *TRPM4* in several human tissues, suggesting that this variant may affect the *TRPM4* gene expression. The increased expression of TRMP4 could aggravate the cation inward flow into the cells of the nervous system [24]. In a situation of relatively mild cerebral hypoxia, e.g., due to insufficient respiration, this increased inflow might mean the difference between a self-limited disorder and manifest and potentially lethal brain edema. However, this locus was not found to be associated with specific SIDS subsets in the following stratified analysis, suggesting a more general effect. It is possible that SIDS risk factors interacting with this locus were not included in the stratified analysis, such as maternal smoking and maternal alcohol consumption. In previous studies on cerebral edema in SIDS, it was reported that maternal smoking in conjunction with some genetic risk factors showed a borderline association with SIDS, and SIDS cases with maternal alcohol consumption were observed with increased weight ratios of brain to body [9, 12]. Regrettably, maternal smoking and alcohol consumption, as possible co-acting factors, were not included in the present study.

Regarding the 8 SNP loci with statistical significance in the stratified analysis, the locus of greatest interest was rs4148622 (*ABCC8*), as it was associated with SIDS in multiple stratified analyses. It was reported by Jha et al. that the wild allele at this SNP could be used for predicting 3-month outcomes of cerebral edema after severe TBI [28], indicating that this polymorphism might be associated with the development of brain edema. The effect allele G of rs4148622 was linked to higher mRNA levels of *ABCC8* in the brain tissues according to the eQTL analysis from the GTEx database. Therefore, one would expect to find an accumulation of the G allele in SIDS. However, the opposite was the case: the frequency of the G allele was lower in SIDS. Nevertheless, higher mRNA levels do not necessarily indicate increased protein expression, as the probe used for the eQTL analysis might just have recognized one splicing isoform: Rs4148622 and its proxy loci (rs2355017 and rs4757516) act as possible splicing variants and might influence the spatial structure of the SUR1 protein, possibly impairing the assembly of the subunits of the SUR1-TRPM4 complex [28]. Considering this, the low frequency of the G allele in SIDS fits well to the hypothesis of SIDS and genetically determined brain edema as postulated by Opdal et al. [11–13].

For rs7105832 and rs3819521 in *ABCC8* that were also associated with subgroups of SIDS in the present study, several studies demonstrated a relation to cerebral edema or increased intracranial pressure [28, 29]. It was demonstrated that the variant allele of rs7105832 was associated with increased average intracranial pressure (ICP) in cerebral edema patients [28], and that the rare homozygous genotype of rs3819521 was linked to increased odds of CT edema after TBI [29].

Interestingly, the *ABCC8* locus and specifically the variant rs3758953 in this study have been reported to be associated with type 2 diabetes, and the carriers of the risk genotypes of rs3758953 had higher levels of both glucose and insulin [32]. It has been shown that hyperglycemia causes the outflow of intracellular water into the tissue interstitial space and thus edema [33].

Besides a potential role in brain edema, SUR1 and TRPM4 as important ion channels are expressed in several human organs such as the heart, brain, and pancreas. Therefore, their physiological roles are diverse and thus could contribute to the individual SIDS risk in more ways than just via brain edema: Loss-of-function variants in *ABCC8* or *TRPM4* can cause lethal cardiovascular and metabolic genetic disorders, such as congenital hyperinsulinism, Brugada syndrome, or long QT syndrome (LQTS), potentially leading to sudden infant death instead of genuine SIDS [34–39]. In a recent study, 2 of 45 victims from sudden unexplained death (in adults) were reported to show a deletion in the coding region of the TRPM4 gene [40]. In a polygenic context for SIDS etiology, multiple genetic and external risk factors may contribute to SIDS simultaneously rather than one genetic variant leading to accepted genuine SIDS. It should, however, be stressed that the specific loss-of-function variants causing the above-mentioned lethal genetic disorders were not studied in the present study.

Some of the variants in genes *ABCC8* (rs7950189, and rs7112138) and *TRPM4* (rs11083962) studied herein reached significant results in different subgroups; however, no previous studies reporting their related association with other diseases could be found. Nevertheless, for these loci was demonstrated by the eQTL analysis that they influence gene expression and thus may make carriers of these loci more susceptible to SIDS. The haplotype analysis demonstrated that the haplotypes in *ABCC8* and *TRPM4*, consisting of risk alleles of partial SNPs, were associated with SIDS. Although the frequency of risk haplotypes is low (< 5%), the results suggest that variants may combine to modulate the risk of SIDS.

Several potential limitations in this study should be mentioned. Firstly, all nominal significant results, after multiple testing corrections, no longer reached the required level of significance (although rs4148622 came close). Therefore, future studies involving larger sample sizes should be performed to validate our findings. Secondly, as limited information on their biological functions is available for some loci tested herein, it is needed to be careful when interpreting their potential roles in SNP-mediated SIDS etiology. Thirdly, some well-known SIDS risk factors linked to cerebral edema like maternal smoking and alcohol consumption have not been included in the present study as related records were not available to us.

In summary, we investigated for the first time the association of genetic variants involved in the SUR1-TRPM4 non-selective cation channel genes related to brain edema with SIDS. Despite these promising results, further studies with a larger sample size are supposed to confirm our results. Even more so, it would be informative if functional studies are conducted in the future to determine the function of risk genotypes and to be better able to interpret their impact on the pathogenesis of SIDS.

## Supplementary Information

Below is the link to the electronic supplementary material.

Supplementary file1 (DOCX 19 kb)

Supplementary file2 (DOCX 31 kb)
