# Supplementary material for: Variants in genes encoding the SUR1-TRPM4 non-selective cation channel and sudden infant death syndrome (SIDS): potentially increased risk for cerebral edema
Source: Int J Legal Med. 2022 Apr 26;136(4):1113–20. doi: 10.1007/s00414-022-02819-9 (PMC9170623; doi:10.1007/s00414-022-02819-9)
Supplement: Supplementary file 1 — Supplementary file1 (DOCX 19 kb) [file 414_2022_2819_MOESM1_ESM.docx]

| **Supplementary material 1** Detailed information of 24 SNPs included in this study | | | | | | | |
| --- | --- | --- | --- | --- | --- | --- | --- |
| SNP | Type | Gene | Position | European MAF | Related article | Association with disease | eQTL |
| rs1048099 | G>A | *ABCC8* | non coding transcript variant, genic upstream transcript variant, synonymous variant, 5 prime UTR variant, coding sequence variant | A=0.480 | Screening for mutations in ABCC8 and KCNJ11 genes in Saudi persistent hyperinsulinemic hypoglycemia of infancy (PHHI) Patients | Persistent Hyperinsulinemic Hypoglycemia of Infancy | None |
| rs10766397 | T>C | *ABCC8* | intron_variant | C=0.375 | None | None | Yes |
| rs11024286 | G>A | *ABCC8* | intron variant | A=0.360 | Downstream TRPM4 polymorphisms are associated with intracranial hypertension and statistically interact with ABCC8 polymorphisms in a prospective cohort of severe traumatic brain injury | Intracranial Hypertension | None |
| rs1799857 | G>A | *ABCC8* | synonymous variant, 5 prime UTR variant, coding sequence variant | A=0.460 | ABCC8 Single Nucleotide Polymorphisms are associated with cerebral edema in severe TBI | odds of decompressive craniotomy | Yes |
| rs1799859 | C>T | *ABCC8* | synonymous_variant,non_coding_transcript_variant,coding_sequence_variant | T=0.291 | Metabolic control in type 2 diabetes is associated with sulfonylurea receptor-1 (SUR-1) but not with KCNJ11 polymorphisms | type 2 diabetes mellitus | Yes |
| rs2283258 | C>T | *ABCC8* | intron variant | T=0.312 | ABCC8 Single Nucleotide Polymorphisms are associated with cerebral edema in severe TBI | brain edema | Yes |
| rs2283261 | A>C | *ABCC8* | Intron Variant | C=0.403 | Downstream TRPM4 Polymorphisms are associated with intracranial hypertension and statistically interact with ABCC8 polymorphisms in a prospective cohort of severe traumatic brain injury | None | Yes |
| rs3758953 | A>G | *ABCC8* | upstream_transcript_variant | G=0.497 | Gene x lifestyle interactions in type 2 diabetes mellitus and related traits | type 2 diabetes mellitus | None |
| rs3819521 | C>T | *ABCC8* | intron variant, genic upstream transcript variant | T=0.351 | ABCC8 Single Nucleotide Polymorphisms are associated with cerebral edema in Severe TBI | Cerebral Edema | Yes |
| rs4148622 | G>A | *ABCC8* | intron variant | A=0.260 | Regionally clustered ABCC8 polymorphisms in a prospective cohort predict cerebral oedema and outcome in severe traumatic brain injury | predicted 3-month outcome | Yes |
| rs60105962 | T>C | *ABCC8* | intron variant | C=0.405 | None | None | Yes |
| rs7105832 | A>C | *ABCC8* | Intron Variant | C=0.351 | Regionally clustered ABCC8 polymorphisms in a prospective cohort predict cerebral oedema and outcome in severe traumatic brain injury | intracranial pressure | Yes |
| rs7112138 | G>A | *ABCC8* | downstream transcript variant | A=0.373 | None | None | Yes |
| rs7950189 | C>T | *ABCC8* | Intron Variant | T=0.454 | None | None | None |
| rs985136 | G>C | *ABCC8* | intron_variant,genic_upstream_transcript_variant,upstream_transcript_variant | C=0.489 | None | None | Yes |
| rs11083962 | T>G | *TRPM4* | genic upstream transcript variant, intron variant | G=0.486 | None | None | None |
| rs11083963 | A>G | *TRPM4* | genic upstream transcript variant, intron variant | G=0.453 | Single nucleotide polymorphisms and genotypes of transient receptor potential ion channel and acetylcholine receptor genes from isolated B lymphocytes in myalgic encephalomyelitis/chronic fatigue syndrome patients | myalgic encephalomyelitis/chronic fatigue syndrome | Yes |
| rs11667393 | A>G | *TRPM4* | genic upstream transcript variant, intron variant | G=0.283 | Downstream TRPM4 Polymorphisms are associated with intracranial hypertension and statistically interact with ABCC8 polymorphisms in a prospective cohort of severe traumatic brain injury | No association | None |
| rs12980226 | A>C | *TRPM4* | genic upstream transcript variant, intron variant | C=0.321 | None | None | Yes |
| rs34271662 | A>G | *TRPM4* | intron variant | G=0.347 | None | None | Yes |
| rs3760662 | A>G | *TRPM4* | upstream transcript variant | G=0.443 | None | None | None |
| rs4802581 | T>C | *TRPM4* | genic upstream transcript variant, intron variant | C=0.450 | None | None | Yes |
| rs7251160 | C>T | *TRPM4* | intron variant | T=0.333 | None | None | Yes |
| rs8104571 | C>T | *TRPM4* | Intron Variant | T=0.050 | Downstream TRPM4 Polymorphisms are associated with intracranial hypertension and statistically interact with ABCC8 polymorphisms in a prospective cohort of severe traumatic brain injury | Intracranial Hypertension | Yes |
