# Supplementary material for: Variants in genes encoding the SUR1-TRPM4 non-selective cation channel and sudden infant death syndrome (SIDS): potentially increased risk for cerebral edema
Source: Int J Legal Med. 2022 Apr 26;136(4):1113–20. doi: 10.1007/s00414-022-02819-9 (PMC9170623; doi:10.1007/s00414-022-02819-9)
Supplement: Supplementary file 2 — Supplementary file2 (DOCX 31 kb) [file 414_2022_2819_MOESM2_ESM.docx]

| **Supplementary material 2.** SNPs with relevant alleles and genes as well as primer and probe sequences | | | | | | | |
| --- | --- | --- | --- | --- | --- | --- | --- |
| SNP | Gene | ASP1 allele | ASP2 allele | ASP1 | ASP2 | LSP | STA |
| rs1048099 | *ABCC8* | A | G | GCACATGGCTTCATTTCCCT | GCACATGGCTTCATTTCCCC | GCAGCATGAAGGTCAGGATCCA | GGTGCACATCCACCACAG |
| rs10766397 | *ABCC8* | C | T | AGCCAGGTATGGTGGCG | TTAGCCAGGTATGGTGGCA | CCTGAGTAGCTGGGACCACA | CAACATGGTGAAACCCTATCTCTAT |
| rs11024286 | *ABCC8* | A | G | GCATTTTGAGGTGCACGGA | GCATTTTGAGGTGCACGGG | TTGACTGCACTGACTATTGGACTGA | GCAAGATGCATCTGCAGGA |
| rs1799857 | *ABCC8* | C | T | GCCTCTTTGAAGAAGCTGACG | GGCCTCTTTGAAGAAGCTGACA | GCCTCACTTGTGCCTCTGTC | CCACGGAGGGCGAGAA |
| rs1799859 | *ABCC8* | A | G | CTCCAACTCCCTGCACAGA | TCCAACTCCCTGCACAGG | GGGCGTAGGTAAGGCCCA | GCAGCGGTGACCTCCAT |
| rs2283258 | *ABCC8* | A | G | AGGGACAAGAGGAGGGAACA | GGGACAAGAGGAGGGAACG | TTCTCACCAATGGGATGCGAGA | AACTAGCCAGAAGAAGGGACA |
| rs2283261 | *ABCC8* | G | T | ACCATGGTCTAGCTGGACAC | CACCATGGTCTAGCTGGACAA | GGCTGGCCATGAATACCCC | CCTGCCTGAGGCATAGGAC |
| rs3758953 | *ABCC8* | C | T | CAGTCAGGGGAACACAAAAGG | ACAGTCAGGGGAACACAAAAGA | GCAGGCTCAGTGAAGCTTGT | AGCATAAATGCACATGTTCTTAGGAA |
| rs3819521 | *ABCC8* | A | G | GGACAGCCTTCAGCTGTGA | GGACAGCCTTCAGCTGTGG | CCAGCTCAGGCTGGTCTCTAG | CTGTCCGGCAGAGTGGA |
| rs4148622 | *ABCC8* | C | T | TCCTTGTGGTCCCAAGAAACG | TCCTTGTGGTCCCAAGAAACA | GCAGCCATGAGCAAAGTATGGA | AGTATGAGCCCAGGCTCC |
| rs60105962 | *ABCC8* | C | T | TGGCTCTGACCACATGGC | GTGGCTCTGACCACATGGT | CTGGCCCTCCCAGTCTTGT | GGCATTTGGAATTGACCCTGT |
| rs7105832 | *ABCC8* | A | C | CATTGCTGTTGGGATAAAGGCA | CATTGCTGTTGGGATAAAGGCC | TGAGCCACCGCACCCA | CACACACACACAACTTCCCA |
| rs7112138 | *ABCC8* | A | G | TGGAAACAGGGCCTGTGAA | TGGAAACAGGGCCTGTGAG | GCCCCACCCCTATTACCTCA | GAAGCCTAATCCTACAATGTAACTGT |
| rs7950189 | *ABCC8* | C | T | AGAACATCAGATTCCCTGCATCC | AAGAACATCAGATTCCCTGCATCT | CCCACAGGGACACAGCTAGA | GATGGCATATATGACTGTTCCCA |
| rs985136 | *ABCC8* | C | G | GGGTCACCCCTCCCC | GGGTCACCCCTCCCG | TCCCTCTTCCCTAGGCCCA | CGTGGCGCAGCTTAGG |
| rs11083962 | *TRPM4* | G | T | ACAGGCGTGAGCCACC | ACAGGCGTGAGCCACA | AGAGGCCCATGGAAAGCGA | CGGCCTCCCAGATTGTTG |
| rs11083963 | *TRPM4* | A | G | GCATGAAGCAGCCGGGA | CATGAAGCAGCCGGGG | CTCCTTGGCAGGATACCCCT | AGAAAGCCACAAACAAGTCAGG |
| rs11667393 | *TRPM4* | A | G | GGTCCCGTTTCTCATCTGA | GGTCCCGTTTCTCATCTGG | TTGTTTAATTCTCATAATAACCCCAGGAGATCA | GGGCAAATCATTTCACCTCTCT |
| rs12980226 | *TRPM4* | A | C | GCTCCATATACAACGGCCCTTATT | GCTCCATATACAACGGCCCTTATG | GCCAGGGCCACTTGTGATAC | CATCAATTTCCTGCCTGGCT |
| rs34271662 | *TRPM4* | A | G | CCAAAGTGCTGGGATTACAGGT | CAAAGTGCTGGGATTACAGGC | GCCTTTAAAAGATTTTAACAGGCCGGG | CGATCCACCTGCCTCGG |
| rs3760662 | *TRPM4* | A | G | CCCCCCGGCCCGGA | CCCCCCGGCCCGGG | GGTCCCAGGCCGCGATA | CTCCCCTGTGTGTCTCTCTC |
| rs4802581 | *TRPM4* | C | T | TGTAGTCCCAGCTACTCAGGAG | TGTAGTCCCAGCTACTCAGGAA | AACCTCCGTCTCCCAGGTTC | TGGTGGTACGCGCCT |
| rs7251160 | *TRPM4* | C | T | CGGTCCACCAGTACCAACTG | CGGTCCACCAGTACCAACTA | ACTGAGGCTCCCAAGGTTCAA | CGTGGGTTCCAGTCCCC |
| rs8104571 | *TRPM4* | C | T | TGTCCAGGCTAACTTTGGAGC | GTGTCCAGGCTAACTTTGGAGT | CAAGGGTCAGATGTCAGAGGTCA | TGATGTTAGTTATTTGTGTCCAGGC |
| ASP: allele-specific primer; LSP: locus-specific primer; STA: specific target applification. | | | | | | | |
|  | | | | | | | |
